# Deuteration may reduce the efficacy of dextromethorphan in treating agitation in Alzheimer’s disease

**DOI:** 10.1186/s13195-025-01780-0

**Published:** 2025-06-09

**Authors:** Anton Bespalov, Jina Swartz, Nadine Knowles, Hans J. Moebius

**Affiliations:** EXCIVA GmbH, Hauptstrasse 25, Heidelberg, 69117 Germany

**Keywords:** Neuropsychiatric symptoms, Behavioral and psychological symptoms of dementia, Agitation, Aggression, Clinical development, Drug development

## Abstract

Agitation is one of the most prevalent neuropsychiatric symptoms leading to institutionalization in individuals with Alzheimer’s disease (AD) dementia. It is associated with poor outcomes, including reduced functional ability, reduced quality of life, accelerated disease progression, increased mortality, and significant economic burden. Following an initial report demonstrating the strong efficacy of a combination of dextromethorphan and the CYP2D6 inhibitor quinidine, several follow-up development efforts have explored this approach. Axsome Therapeutics has reported positive results in three out of four clinical trials evaluating AXS-05, a combination of dextromethorphan with another CYP2D6 inhibitor, bupropion. In contrast, Otsuka’s AVP-786, a combination of deuterated dextromethorphan and quinidine, has yielded predominantly negative results. It is widely believed that deuteration alters a molecule’s pharmacokinetic properties without affecting its pharmacodynamics. However, in our patch-clamp experiments, deuteration resulted in a 16-fold increase in IC50 for dextrorphan and about two-fold increase for dextromethorphan at NMDA receptors containing the NR2D subunit. Thus, based on both clinical data and emerging pharmacological evidence, we hypothesize that AVP-786 failed to demonstrate efficacy in treating agitation in AD dementia due to the negative impact of deuteration on dextromethorphan’s pharmacodynamic properties.

Agitation is one of the most common neuropsychiatric symptoms leading to institutionalization in subjects with Alzheimer’s disease (AD) dementia and is associated with a negative prognosis in terms of function, quality of life, disease course, mortality and associated economic costs. In most parts of the world, current therapy options are limited to antipsychotics.

In 2015, Cummings and colleagues reported robust efficacy of AVP-923, a combination of dextromethorphan (30 mg bid; CYP 2D6 substrate) and quinidine (10 mg bid; CYP 2D6 inhibitor), against agitation in AD dementia patients [1], clinicaltrial.gov ID: NCT01584440). These results triggered several follow-up development efforts in this indication. Axsome Therapeutics reported positive results from three out of four clinical studies with AXS-05, a combination of dextromethorphan (45 mg bid) with another CYP 2D6 inhibitor, bupropion (105 mg bid) (NCT03226522; NCT04797715; NCT05557409; NCT04947553; [2]). In contrast, Otsuka reported predominantly negative results for AVP-786, a combination of deuterated dextromethorphan (20, 30 or 45 mg bid) with quinidine (5 mg bid) (NCT02442765, NCT02442778, NCT03393520).

There appears to be no extrinsic factor associated with the conduct of these clinical studies that could readily explain the divergent outcomes: i) the same team (Avanir/Otsuka) delivered both positive and negative results using similar operationalization models; ii) the same primary outcome instrument was used (Cohen-Mansfield Agitation Inventory, CMAI) in studies with positive and negative results; iii) the same study designs were used in positive and negative studies (sequential parallel comparison design [SPCD] and classical parallel-group); iv) positive results were generated by two independent teams (Avanir/Otsuka and Axsome) using two different CYP 2D6 inhibitors (quinidine, bupropion) and three different study designs (SPCD, classic parallel-group and randomized withdrawal). With the results of AXS-05 and AVP-786 remaining unpublished, it is not possible to judge whether there were other potentially confounding factors that should be considered (e.g., patient demographics, disease severity at baseline, strength of placebo response, etc.).

Thus, there exist four positive randomized controlled trials with non-deuterated dextromethorphan and three studies with negative or mixed results with deuterated dextromethorphan. At first sight, this divergence might be puzzling. Until recently, deuteration was often used by biopharma and biotech to generate novel intellectual property for established drugs. The underlying assumption for this approach was that deuteration makes molecules metabolically more stable, while not affecting their pharmacodynamic properties.

On the basis of these beliefs and supported by evidence on lack of changes in the receptor binding profile [3], Avanir/Otsuka conducted a Phase 1 study with AVP-786 to confirm the desired plasma exposures for deuterated dextromethorphan, and then launched a broad Phase 3 program with AVP-786. One may assume that the Phase 1 study established pharmacokinetic bioequivalence between AVP-786 and AVP-923 (the original combination of non-deuterated dextromethorphan and quinidine) in terms of both the exposures achieved for the parent molecule (dextromethorphan) and the profile of all metabolites that were monitored during the AVP-923 program (e.g., dextrorphan produced by CYP 2D6, 3-methoxymorphinan produced via by CYP 3A4/5). In the absence of such publicly accessible information, one cannot fully rule out a hypothetical scenario that deuteration steered metabolism of dextromethorphan in a direction (e.g., greater role of CYP 3A4/5) leading to formation of metabolites such as 3-methoxymorphinan that interfere with the activity of dextromethorphan.

There is, however, a growing body of evidence indicating that deuteration can affect pharmacodynamic properties of molecules in diverse fields (even if three- to five-fold differences in IC_50_ or K_i_ may have limited or no impact on therapeutic efficacy) such as: i) activation of the sweet taste receptor TAS1R2/TAS1R3 by D_2_O but not H_2_O [4],ii) changes in ligand affinity to noradrenaline transporter [5] and adenosine A_2A_ receptor [6], iii) higher affinity and stronger in vivo activity of deuterated heroin vaccine compared to protium heroin vaccine – in vitro and in vivo animal evidence [7], iv) impact of deuterated medium on agonist binding at H_2_ histamine receptors – in silico and in vitro evidence ([8]; v) impact of deuteration on pharmacodynamic properties of GABA_A_ positive allosteric modulators [9].

Thus, it should be considered whether deuteration can be responsible for the clear dissociation between clinical outcomes with deuterated and non-deuterated dextromethorphan in agitation in AD.

Dextromethorphan has multiple mechanisms of action including 5-hydroxytryptamine (5-HT) reuptake inhibition and N-methyl-d-aspartate (NMDA) receptor channel blockade [10]. Dextromethorphan is metabolized primarily by CYP 2D6 to dextrorphan, which retains many of dextromethorphan’s pharmacological properties. It is not known whether therapeutic efficacy of dextromethorphan against agitation in AD dementia is driven by dextromethorphan, dextrorphan or both, nor it is known which receptor mechanism(s) are responsible for these clinical effects. As another NMDA receptor channel blocker, memantine, was previously demonstrated to have effects against agitation in AD [11], this target is discussed as a potential candidate contributing to therapeutic efficacy of dextromethorphan.

It was previously suggested that deuteration attenuated in vivo pharmacodynamic NMDA receptor antagonist properties of dextromethorphan in mice [12]. However, these results could be explained by higher metabolic stability of deuterated dextromethorphan and, therefore, reduced formation of dextrorphan, a more potent NMDA receptor channel blocker than dextromethorphan [10].

We have now generated in vitro patch clamp data comparing NMDA receptor channel blocking properties of dextromethorphan and dextrorphan (deuterated and non-deuterated) at NMDA receptors containing the NR2D subunit. As shown in Fig. 1, deuteration resulted in a 16-fold increase in the half maximal inhibitory concentration (IC_50_) for dextrorphan and about two-fold increase for dextromethorphan. These results confirm that deuteration is not an “innocent” procedure and, contrary to common belief, can affect the pharmacodynamic activity of molecules in various ways.

**Fig. 1:**
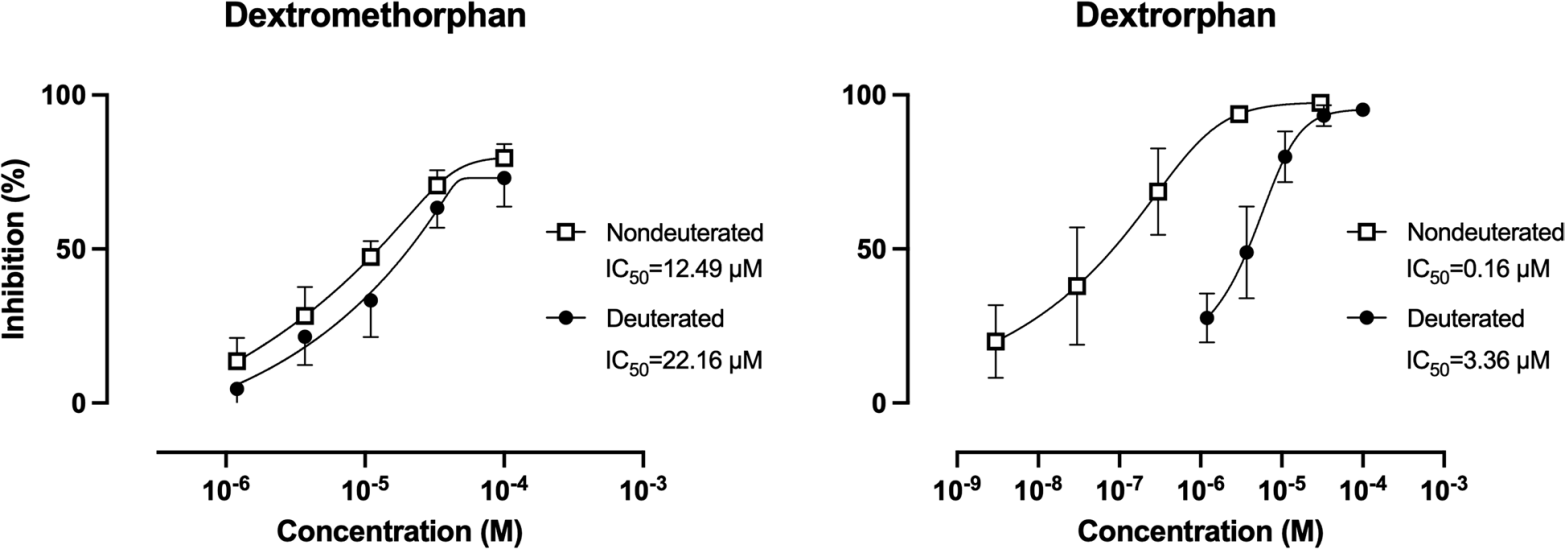
Concentration-dependence of the blockade of human recombinant NR1/NR2D receptors by dextromethorphan and dextrorphan (whole-cell patch-clamp). NR1/NR2D receptors were stably expressed in flpinTREx293 cells (HD Biosciences) maintained in cell culture medium (DMEM + 10% FBS + 1% PS) with antibiotics (800 ug/mL G418, 50 ug/mL Hygromycin B). One day before the experiment, doxycycline (1 ug/mL) was added to culture medium to induce the expression of NR1A. For electrophysiological recording, cells were seeded on glass cover slips coated with poly-L-ornithine and incubated for 1–24 h in 35 mm dishes before being transferred to the recording chamber. The recording chamber was continuously perfused (1–1.5 ml/min) with extracellular solution (137 mM NaCl, 1.8 mM CaCl2, 4.0 mM KCl, 10 mM Glucose, 10 mM HEPES; pH adjusted to 7.4 with 2M NaOH). The electrodes had resistance of ~ 3–5 MΩ and were filled with a solution containing 140 mM CsCl, 10 mM HEPES, 10 mM EGTA (pH adjusted to 7.25 with 1M CsOH). The electrophysiological responses were obtained using the conventional voltage-clamp method (HEKA EPC10USB amplifier). A holding potential of −70 mV was used for all assays. Peak current responses (i.e., at the end of application of 20 μM glycine + 80 μM NMDA) were normalized to control levels and plotted as means (± standard deviation) against drug concentration (*n* = 3–7 per concentration). Estimation of half maximal inhibitory concentrations (IC_50_) and curve fitting were made according to the logistic equation (GraphPad Prism)

Previous studies have established that administration of CYP 2D6 inhibitors results in the increase of plasma concentration of dextromethorphan accompanied by the decrease in concentration of its main metabolite dextrorphan. With strong inhibitors such as quinidine (as in AVP-923 or AVP-786), plasma concentration of dextromethorphan may increase more than 20-fold while concentration of total dextrorphan (glucuronidated and nonglucuronidated) is reduced by more than ten-fold. Interestingly, under the same conditions of CYP 2D6 inhibition, the concentration of free (non-glucuronidated) dextrorphan is reduced only by about 20–30% [13]. As it is the free (non-glucuronidated) dextrorphan that readily crosses the blood–brain barrier, free dextrorphan likely continues to contribute to the net effects of dextromethorphan even under conditions of CYP 2D6 inhibition. If so, a significant reduction in NMDA receptor antagonist potency by deuterated dextrorphan can alter the overall pharmacodynamic profile of dextromethorphan.

Dextromethorphan is a pluripotent drug and the NMDA receptor is only one of its targets. It is not known whether NMDA receptor antagonist properties of dextromethorphan or dextrorphan are required for dextromethorphan’s efficacy in people with AD dementia. It is also unknown whether deuteration affects the ability of dextromethorphan (or dextrorphan) to interact with targets other than the NMDA receptor. One can only hypothesize, concluding from both clinical data and emerging pharmacological evidence presented here, that AVP-786 lacked efficacy against agitation in AD dementia because of a negative impact of deuteration on the pharmacodynamic properties of dextromethorphan and/or its metabolites.

## Data Availability

The datasets used and/or analyzed during the current study are available from the corresponding author on reasonable request.
